# Dr. Nagle on the Diagnosis of Pregnancy

**Published:** 1831-04-01

**Authors:** 


### DUBLIN LYING-IN HOSPITAL.

### ON THE DIAGNOSIS OF PREGNANCIES. By Dr. NAGLE.

DR. N. has published two cases in the Lancet, where the existence of twins was clearly ascertained by auscultation, sometime before parturition. One of the cases will be a sufficient illustration.

*Case.* "On the 15th October, a female, aged about 30, and in her first pregnancy, was admitted into the Lying-in Hospital, Dublin. The abdomen was, in this case, so enlarged as to lead to the suspicion of twins; and on the next morning my attention was directed to the patient by one of the nurses. Having applied the stethoscope, with a sheet interposed between its sternal extremity and the abdomen, I found a fœtal heart to pulsate strongly, rapidly, and rather irregularly, midway between the umbilicus and the *superior* anterior spinous process of the left ilium. By a minute examination I satisfied myself that the cylinder was applied immediately over the fœtal heart, with the rhythm of which I took particular care to make myself familiar. I next directed my attention to the other parts of the abdomen, still hearing the pulsations of a fœtal heart, until I came on a point where they were most distinctly audible. This greater distinctness of resonance I found to be nearly under the linea semilunaris, between the umbilicus and the anterior inferior spinous process of the *right* ilium. The pulsations here I immediately recognised to be weaker, less rapid, and less regular in rhythm, varying from 125 to 133 in a minute, whilst those on the left side varied from 160 to 170. The patient, labouring under a smart bronchitis, was occasionally attacked with a severe fit of coughing, during which, the abdomen receiving a strong concussion, the pulsations of the fœtal heart, on the right side, were remarkably accelerated, whilst those on the left were scarcely at all affected.

In order to draw a diagnosis, I compared, with as much accuracy as I was capable, the pulsations on both sides with each other, and then each separately with the impulse at the chest, and the pulsations at the wrist, of the mother. The diagnosis was, that there were twins; and I may add, that auscultation induced me to predict, that the head of the second child would present.

The announcement of this discovery was received with considerable interest by some whom I took to examine the case; and Dr. Collins, the highly respectable master of the Hospital, was so satisfied of the accuracy of the diagnosis, that he declared 'he could no longer repose confidence in the stethoscope in the practice of midwifery if the case did not prove to be twins.' The patient, owing to a want of action in the uterus, continued to suffer a tedious, and at times a distressing, labour, until the night of the 20th, when after the use of 45 grains of the ergot of rye, in divided doses, which at first quickened, then lowered the pulse, and evidently soon produced some slight action in the uterus, she was delivered of twins, the *heads* of both presenting, the delivery of the second being assisted with the forceps. From the nature and length of the woman's labour both children were dead; the second exhibiting the appearance of having been alive a short time previous to birth. The placenta in this case was single, and had to be removed by art."

In another case he was able to detect the existence of twins, though the female was confident that her child was dead, as she had ceased to feel its motions. As these diagnoses were delivered in a public hospital, there can be no doubt of their authenticity.

